# Cardiovascular Outcome of the SGLT2 Inhibitor in Acute Myocardial Infarction: A Meta-Analysis

**DOI:** 10.31083/RCM26136

**Published:** 2025-02-19

**Authors:** Siqi Hu, Ting Tang, Qingwen Yu, Xuhan Tong, Yao You, Shenghui Zhang, Chen Chen, Jiake Tang, Chunyi Wang, Hu Wang, Xinyan Fu, Juan Chen, Xingwei Zhang, Mingwei Wang, Ling Liu

**Affiliations:** ^1^Department of Cardiology, Affiliated Hospital of Hangzhou Normal University, Zhejiang Key Laboratory of Medical Epigenetics, School of Basic Medical Sciences, Hangzhou Institute of Cardiovascular Diseases, Engineering Research Center of Mobile Health Management System & Ministry of Education, Hangzhou Normal University, 310015 Hangzhou, Zhejiang, China; ^2^Department of Cardiology, Hangzhou Lin’an Fourth People’s Hospital, 311321 Hangzhou, Zhejiang, China

**Keywords:** cardiovascular outcome, SGLT2 inhibitor, acute myocardial infarction, meta-analysis

## Abstract

**Background:**

Unexpected cardiovascular events are likely to occur within a short period following an acute myocardial infarction (AMI). The sodium-glucose co-transporter 2 inhibitor (SGLT2-I) is a recently recommended drug for the treatment of AMI. However, its role in the risk of the outcomes following an AMI, including all-cause death and heart failure readmission, remains controversial. Therefore, in this study, we explored the effect of SGLT2-Is on cardiovascular outcomes after an AMI.

**Methods:**

PubMed, Web of Science, and Embase were searched without language restrictions to retrieve case-control studies published before April 2024. Citations were independently screened by two authors, and the studies meeting the predefined inclusion criteria were retained. Data on author names, year of publication, location of the study group, gender and age of participants, outcome assessment, adjusted odds ratios (ORs) and 95% confidence intervals (CIs), and the follow-up period were extracted.

**Results:**

Eight studies were eligible for inclusion, and these studies showed that the use of SGLT2-Is after an AMI was significantly associated with a lower risk of hospitalization for heart failure (OR: 0.66, 95% CI 0.57–0.76, *p* < 0.01) and a lower incidence of major cardiovascular adverse events (OR: 0.79, 95% CI 0.70–0.89, *p* < 0.01), but was unrelated to a lower incidence of all-cause mortality (OR: 0.84, 95% CI 0.69–1.02, *p* = 0.07).

**Conclusions:**

Compared with placebo, SGLT2-I therapy following an AMI can reduce the risk of heart failure hospitalization and the incidence of major cardiovascular adverse events, but has no effect on all-cause mortality.

**The PROSPERO registration:**

CRD42024542335, https://www.crd.york.ac.uk/prospero/display_record.php?ID=CRD42024542335.

## 1. Introduction

Acute myocardial infarction (AMI) is still one of the leading causes of death worldwide [1]. Even in the current era, the 30-day mortality rate and readmission rate of older patients with an AMI in the United States after 10 days of hospitalization is greater than 70% and 25%, respectively [2].

New therapies for myocardial infarction (MI) have been gradually implemented in recent years. These therapies are effective and meet the requirements of guidelines. However, survivors of an AMI still face the risk of unexpected cardiovascular events after infarction, which include both fatal and non-fatal events. Among these risks, heart failure (HF) is the most common complication after myocardial infarction and the strongest predictor of mortality. Therefore, it is of great significance to solve the problem of heart failure for the treatment of patients with an AMI [3].

Sodium-glucose co-transporter 2 inhibitors (SGLT2-Is) is an oral hypoglycemic drug, which has been shown to effectively control blood sugar in patients with type 2 diabetes mellitus (T2DM) [4]. Studies have shown that SGLT2-Is not only significantly improves the cardiovascular and renal health of patients with T2DM, but also has positive therapeutic effects in non-diabetic individuals with or without HF [5, 6, 7]. The “Guidelines for Diagnosis and Treatment of Heart Failure in China 2024” [8] recommend that patients with HF with a reduced ejection fraction (HFrEF) should receive the “new quadruple” treatment scheme as soon as possible with a small dose and incorporate SGLT2-Is into the original “golden triangle” treatment. Studies have shown that adding SGLT2-Is to the “golden triangle” treatment can significantly reduce the risk of all-cause death and cardiovascular death in patients with HFrEF by 13% and 14%, respectively [9].

However, a recent EMPACT-MI trial [10] has shown that in patients with an AMI, the risk of the composite primary endpoint (first hospitalization due to HF or death from any cause) did not decrease significantly in AMI patients. Therefore, the purpose of the present study is to review the existing literature to determine the role of SGLT2-Is on the prognosis of patients with an AMI.

## 2. Methods

### 2.1 Search Strategy and Eligibility Criteria

The protocol for this study was conducted based on the Preferred Reporting Items for Systematic Reviews and Meta-Analyses (PRISMA) statement. We searched the related research published before April 2024 on PubMed, Embase and Web of Science. The studies to be included for this meta-analysis were determined by using various combinations of the following search words: SGLT2-Is, myocardial infarction or acute myocardial infarction. There were no language restrictions in the search strategy. In addition, we also searched the abstracts of scientific conferences and references.

The retrieved citations were screened by two reviewers (SQH and YY) independently reading the titles and abstracts, and the qualified articles were reviewed. The criteria for inclusion in this study were as follows: (1) Participants were AMI patients; (2) The study compared the intervention group (SGLT2-Is group) and the control group; (3) Odds ratios (ORs) and 95% confidence intervals (CIs) were provided to compare the efficacy of intervention and control groups in treating patients with an MI. The exclusion criteria were: (1) The study was a review or animal study; (2) The reported efficacy evaluation indicators were only laboratory indicators or echocardiographic results.

### 2.2 Data Extraction and Quality Assessment

The following basic information were extracted independently by the authors from each study: author, year of publication, study group location, gender and age of target participants, outcome assessment, adjusted or and 95% CI, and follow-up period. The quality of the studies obtained from the literature search according to the PROSPERO reporting guidelines were assessed by two reviewers [11]. Quality evaluation was determined by: (1) The inclusion and exclusion criteria; (2) Records of follow-up and loss of follow-up; (3) Clear definition of and evaluation of the results; (4) Sufficient follow-up time; (5) Appropriate statistical analysis; (6) Identification of important confounders and prognostic factors. A score of 1 is given when a criterion is met, 0 is given if the criterion is unclear, and –1 is given if it is not met.

### 2.3 Statistical Analysis

The Review Manager 5.4 (RevMan Development Core Team, Oxford, England) and Stata 12 (StataCorp LP, College Station, TX, USA) were used for statistical analysis. Before pooling the data, the adjusted OR for each study was converted to logOR to stabilize the variance and standardize the distribution. The standard error (SE) of logOR was calculated based on a reported 95% confidence interval. The χ^2^ test was used to assess clinical heterogeneity among individual study estimates and quantified with the *I*^2^ statistic. Heterogeneity was divided into 25%, 50%, and 75% corresponding to low, medium, and high heterogeneity, respectively, and sensitivity analysis consisted of sequentially excluding one study to assess its impact on the overall effect estimate. Funnel plots and Egger test were used to evaluate the presence of publication bias. A two-sided value of *p* < 0.05 was considered significant statistically.

## 3. Results

### 3.1 Search Results

**Fig. 1.**
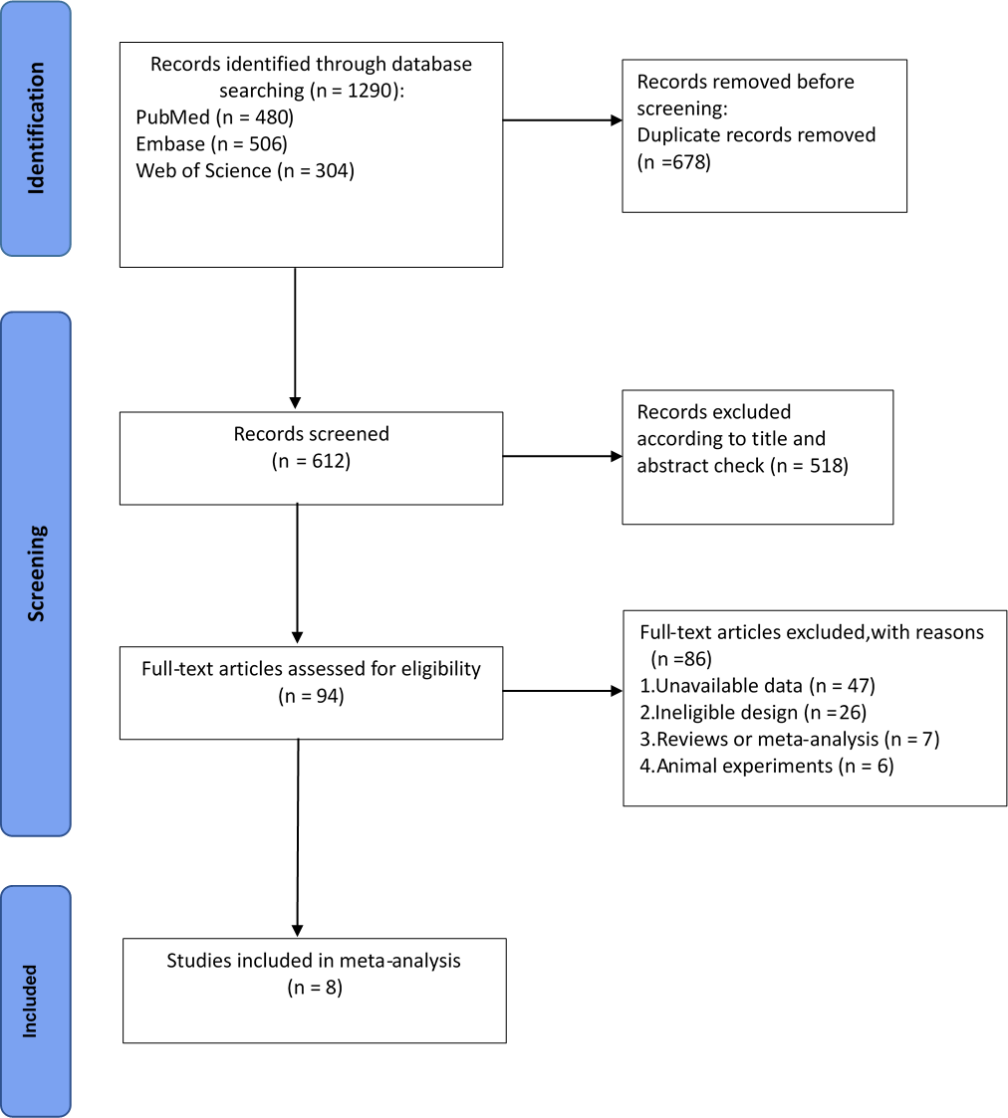
**Flow diagram of the study selection process**.

The PRISMA flow chart describing the process of document retrieval is shown in Fig. 1. A combination of electronic and manual searches produced a total of 1290 potentially relevant publications. After an initial selection of titles and abstracts, 518 papers were excluded. After the full text review, 86 additional articles were excluded due to (1) substandard design, (2) lack of available outcome data, or (3) studies design as a review, a meta-analysis or an animal study; Ultimately, eight unique cohort studies [10, 12, 13, 14, 15, 16, 17, 18] were identified as eligible for inclusion in the meta-analysis.

### 3.2 Characteristics of the Included Studies

The characteristics of the individual studies are presented in Table 1 (Ref. [10, 12, 13, 14, 15, 16, 17, 18]). The table summarizes data from eight studies, involving a total of 32,859 participants, regarding the effects of SGLT2-Is on cardiovascular events following an AMI. These events encompassed hospitalization due to HF, major adverse cardiac events (MACE), and all-cause mortality, as reported by eight [8, 10, 11, 12, 13, 14, 15, 16], five [10, 11, 13, 15, 16], and five [8, 10, 11, 12, 13] studies, respectively. Follow-up durations ranged from a median of 0.97 to 5.4 years. The majority of participants were male.

**Table 1.** **Characteristics of studies contained in the meta-analyses**.

Name, year	Types of studies	Country	Participant (man, %)	Intervention drug	Outcome	Age mean (SD)	Follow-up (year)	Quality score
Butler *et al*. [10], 2024	RCT	UK	6522 (75%)	Engagliflozin 10 mg	All-cause death, hospitalization for heart failure	63.65	1.49	6
James *et al*. [13], 2024	RCT	Sweden	4017 (79.9%)	Dapagliflozin 10 mg	Hospitalization for heart failure, MACE, all-cause death	62.9	0.97	6
Furtado *et al*. [12], 2019	RCT	USA	17,160 (37.4%)	Dapagliflozin 10 mg	Hospitalization for heart failure, MACE, all-cause death	63	5.4	6
Paolisso *et al*. [17], 2023	Retrospective study	Italy	646 (77.1%)	SGLT2-I	Hospitalization for heart failure, MACE, Recurrent AMI	70	2	4
Kwon *et al*. [14], 2023	Retrospective study	Korea	2814 (79.9%)	SGLT2-I	Hospitalization for heart failure, MACE, all-cause death	57.2	2.1	4
Zhu *et al*. [18], 2022	Retrospective study	China	786 (76.5%)	Dapagliflozin	Hospitalization for heart failure, MACE	62 ± 14	1.92	5
Mao *et al*. [16], 2023	Retrospective study	China	462 (78.3%)	Dapagliflozin	Hospitalization for heart failure, MACE	-	1.47	5
Liu *et al*. [15], 2024	Retrospective study	China	452 (61.4%)	SGLT2-I	Hospitalization for heart failure, MACE, all-cause death	-	1	4

SGLT2-I, sodium-glucose co-transporter 2 inhibitor; MACE, major adverse cardiac events; RCT, randomized controlled trial; AMI, acute myocardial infarction.

### 3.3 Incidence of Hospitalization for HF and MACE

**Fig. 2.**
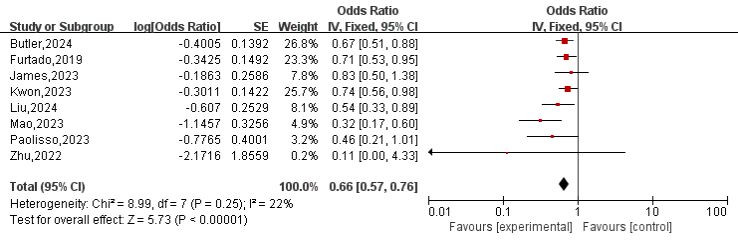
**Forest plot of heart failure hospitalization for patients on SGLT2-I therapy after AMI compared to placebo group**. SGLT2-I, sodium-glucose co-transporter 2 inhibitor; AMI, acute myocardial infarction; CI, confidence interval; IV, inverse variance; SE, standard error.

**Fig. 3.**
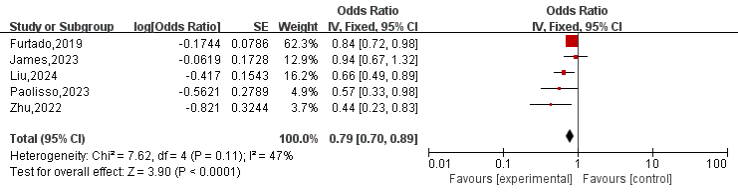
**Forest plot of MACE for patients on SGLT2-I therapy after AMI compared to placebo group**. SGLT2-I, sodium-glucose co-transporter 2 inhibitor; AMI, acute myocardial infarction; CI, confidence interval; MACE, major adverse cardiac events; IV, inverse variance; SE, standard error.

Eight [8, 10, 11, 12, 13, 14, 15, 16] studies reported outcomes on hospitalization for HF. As illustrated in Fig. 2, the overall OR for hospitalization due to HF was 0.66 (95% CI 0.57–0.76, *p* < 0.01) in a fixed-effects model, with low heterogeneity (*I*^2^ = 22%; *p* = 0.25). Additionally, five [10, 11, 13, 15, 16] studies reported outcomes on MACE. As depicted in Fig. 3, the overall OR for MACE was 0.79 (95% CI 0.70–0.89, *p* < 0.01) in a fixed-effects model, with low heterogeneity (*I*^2^ = 47%; *p* = 0.11).

### 3.4 Incidence of All-Cause Death

**Fig. 4.**
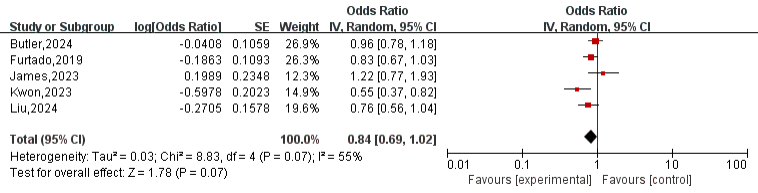
**Forest plot of all-cause death for patients on SGLT2-I therapy after AMI compared to the placebo group**. SGLT2-I, sodium-glucose co-transporter 2 inhibitor; AMI, acute myocardial infarction; CI, confidence interval; IV, inverse variance; SE, standard error.

Five [8, 10, 11, 12, 13] studies provided data on all-cause mortality. As depicted in Fig. 4, the overall OR for all-cause death was 0.84 (95% CI 0.69–1.02, *p* = 0.07) in a random-effects model. No statistically significant difference (*p* > 0.05) was observed between the two groups.

### 3.5 Publication Bias

**Fig. 5.**
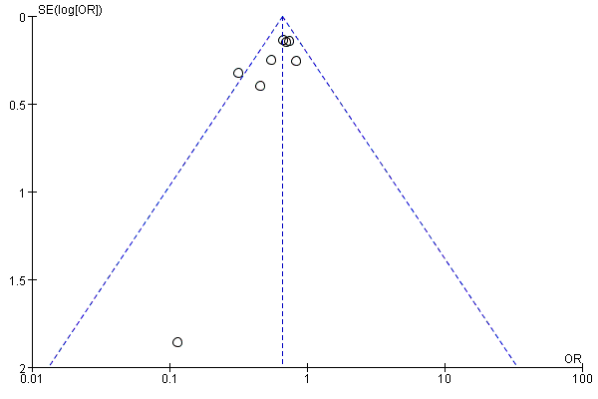
**Filled funnel plot of results of eight studies based on the result of hospitalization for heart failure**. OR, odds ratio; SE, standard error.

The funnel plots are presented in Fig. 5. The funnel chart is symmetrical as a whole, indicating that there is no large publication deviation. But the Egger’s test (*p* = 0.008) showed that there was a significant publication bias.

### 3.6 Sensitivity Analysis

Sensitivity analysis suggests that a study by Mao *et al*. [16] may be the source of heterogeneity in heart failure hospitalization rates. After excluding the Mao *et al*. study [16], the heterogeneity was reduced to *I*^2^ = 0%. Additionally, the exclusion of any single study associated with all-cause mortality or cardiovascular events did not significantly alter the pooled OR or heterogeneity.

## 4. Discussion

SGLT2-Is can not only improve the prognosis of patients with HF, but also improve the cardiovascular prognosis of patients with T2DM and chronic kidney disease (CKD) [19, 20]. However, the recent EMPACT-MI study [10] indicated that compared with placebo, treatment with empagliflozin did not significantly reduce the risk of initial hospitalization for HF or all-cause mortality in patients at an increased risk of HF following an AMI. Our meta-analysis of eight [8, 10, 11, 12, 13, 14, 15, 16] case-control studies provides additional evidence that SGLT2-Is can reduce the readmission of HF and the incidence of major adverse cardiovascular events in patients with AMI. But, the improvement effect of SGLT2-Is on all-cause mortality was not statistically significant.

Considering the dual pressures of an aging population and increasing metabolic risk factors, the burden of cardiovascular diseases is expected to continue to increase. This underscores the need for new approaches for disease prevention, treatment, and medical resource allocation. Early management of pathological changes and timely interventions to improve the prognosis of cardiovascular survivors are becoming increasingly important [21].

Patients with an AMI not only have poor long-term prognosis, but also a significant increase for the risk of adverse endpoint events. These adverse events include nonfatal MI, nonfatal stroke, all-cause death, HF and emergency coronary revascularization [22]. The plan to treat heart failure after MI was initiated more than ten years ago. These include early reperfusion therapy for ST-segment elevation myocardial infarction (STEMI), early application of angiotensin converting enzyme inhibitors (ACEI)/angiotensin II receptor blockers (ARB) and mineralocorticoid receptor antagonists (MRA), routine administration of high-dose statins, and early evaluation of the indications for β-blockers during and after a myocardial infarction [23]. Despite significant advancements in modern medicine, particularly emergency percutaneous coronary intervention (PCI), in the treatment of AMI, survivors still face considerable cardiovascular risks. Many patients with AMI experience adverse events shortly after discharge [24].

SGLT2-Is is the latest recommended drug. The results of a large meta-analysis published in the Lancet showed that SGLT2-Is reduced the risk of acute kidney injury by 23% and the risk of cardiovasc ular death or heart failure hospitalization by 23%, and similar results were obtained in diabetic patients and non-diabetic patients. SGLT2-Is also reduced the risk of cardiovascular death (0.86, 0.81–0.92), but did not significantly reduce the risk of non-cardiovascular death (0.94, 0.88–1.02). Similar results in deaths were found in patients with T2DM and non-diabetic patients [25]. Although SGLT2-Is is mainly used as a hypoglycemic agent, Hwang *et al*. [26] has shown that SGLT2-Is can improve left ventricular systolic and diastolic function and left ventricular geometry in diabetic patients regardless of whether heart failure is present. The improvement of left ventricular function in HFrEF patients was significantly greater than that in heart failure with preserved ejection fraction (HFpEF) patients and patients without HF. These effects of SGLT2-Is may help to reduce the incidence and mortality of HF in patients with T2DM [26]. In addition to HF, compared with non-SGLT2-I users, the use of SGLT2-Is in patients with T2DM admitted for an AMI can significantly reduce the inflammatory reaction and infarct area. These beneficial effects are unrelated to the control of glucose metabolism [27]. According to the findings from several large-scale randomized clinical trials, the use of these medications can reduce cardiovascular mortality and the rate of HF hospitalizations [28]. In animal studies, empagliflozin exhibited the ability to mitigate cardiac fibrosis in its initial stages following an AMI, through the inhibition of the transforming growth factor-β1 (TGF-β1)/Smad3 fibrosis pathway, unrelated to the medication’s hemodynamic effects. Pre-administration of canagliflozin in a pig model before coronary artery occlusion resulted in enhanced myocardial performance during ischemic conditions [29]. Inhibition of sodium-glucose co-transporter 2 (SGLT2) can promote urinary sodium excretion and decrease plasma volume, as noted by the decrease of systolic blood pressure by 3–6 mm Hg and diastolic blood pressure by 1–1.5 mm Hg [30]. SGLT2 has been shown to be expressed in proximal renal tubule cells, but not in human cardiomyocytes. Therefore, whether SGLT2-Is has the potential for specific beneficial effects on the cardiovascular system still needs to be determined. SGLT2-Is may increase diuresis or urine sodium, control the blood glucose, lower blood pressure, reduce weight, improve vascular function, and alter the processing of tissue sodium [31]. Other potentially beneficial cardiovascular effects of SGLT2-Is include reducing adipose-tissue and pro-inflammatory cytokine mediated inflammation, inhibiting the sympathetic nervous system, preventing ischemia/reperfusion injury, enhancing cardiac energy metabolism through transfer to ketone bodies, reducing oxidative stress, and inhibiting signal transduction of advanced glycosylation end products [32]. Specifically, SGLT2-Is leads to “fasting mimicry” by increasing urine glucose. This in turn activates enzymes with antioxidant and anti-inflammatory properties, mainly silent mating type information regulation 2 homolog-1 (SIRT1) and adenosine 5’-monophosphate-activated protein kinase (AMPK), which improve heart function [33]. In terms of the influence of cardiac structure and function, SGLT2-Is seems to directly affect the diastolic function. After the treatment with SGLT2-Is, it has been shown that left ventricular mass decreased significantly. It is possible for SGLT2-Is to inhibit negative cardiac remodeling and improve diastolic function by improving endothelial function [34]. Although the exact mechanism is not clear, the immune metabolism mechanism has attracted increasing attention. Therefore, the cardioprotective characteristics of SGLT2-Is may come from two aspects: the direct effect (dependent on hypoglycemia) and the glucose-independent effect.

Liu *et al*. [15] showed that SGLT2-Is is associated with a significant reduction in the risk of cardiovascular disease (CVD), caused by a significant reduction in CVD death and the readmission for HF. This finding is similar to our results, indicating that SGLT2-Is can improve the prognosis following an AMI. SGLT2-Is has become an effective drug to improve the CVD morbidity and mortality in AMI patients [35]. The meta-analysis by Idowu *et al*. [36] also obtained similar results to ours, suggesting that it is related to the reduction of risk of hospitalization for HF, but not to all-cause mortality.

We incorporated eight high-quality articles to more deeply explore the CVD outcomes of SGLT2-Is following an AMI. In our meta-analysis, although we tried to include 8 high-quality documents closely related to the topic, after Egger test, the results showed that there was a significant publication bias. This discovery suggests that some literatures with negative results or low research quality may not be published for various reasons, which leads to the positive results in the included literatures. We have tried and considered the possibility of including more related literature or searching for grey literature. However, due to the limitation of resources and time, we failed to completely solve this problem. But we noticed the heterogeneity of hospitalization rate of HF in Mao *et al*.’s study [16], which may be because they excluded patients who stopped using dapagliflozin from the analysis. In fact, this withdrawal may also be due to the occurrence of cardiovascular adverse events. However, because the median follow-up time is short, the long-term effects of using SGLT2-Is after MI remain unknown. Additionally, three [12, 13, 15] of the included studies did not explicitly mention the names of the intervention drugs. Therefore, future studies using variations of SGLT2-Is may lead to different insights into cardiovascular outcomes. It is possible that the initial time period for the use of SGLT2-I in patients with MI may affect the prognosis.

## 5. Conclusions

Therapy with SGLT2-Is following an AMI can reduce the risk of HF hospitalization and the occurrence of MACE. However, the therapy has no effect on all-cause mortality. Hence, early administration of SGLT2-Is after MI is recommended.

## Availability of Data and Materials

The datasets used and/or analyzed during the current study are available from the corresponding author on reasonable request.

## Supplementary Material

Supplementary material associated with this article can be found, in the online version, at https://doi.org/10.31083/RCM26136.
